# Intravenous versus oral ‘l-ornithine-l-aspartate’ in overt hepatic encephalopathy: a randomized comparative study

**DOI:** 10.1038/s41598-024-62293-8

**Published:** 2024-05-24

**Authors:** Ashok Jhajharia, Shashank Singh, Sangeeta Jana, Prachis Ashdhir, Sandeep Nijhawan

**Affiliations:** 1 Department of Gastroenterology, SMS Medical College & Hospital, Jaipur, Rajasthan 302004 India; 2 House No. 109, Shiv Vihar, VKI, Infront of road number 5, Sikar Road, Jaipur, 302039 India

**Keywords:** Overt hepatic encephalopathy (OHE), l-ornithine-l-aspartate (LOLA), Randomized controlled trial (RCT), Child turcotte pugh (CTP), Diseases, Gastroenterology

## Abstract

Hepatic encephalopathy (HE), a morbid ordeal affecting chronic liver disease patients always insists for the search of a rational, superior & infallible agent beyond the time-proven standards i.e., Lactulose & Rifaximin. In this RCT, we compared the efficacy of intravenous (IV) l-ornithine-l-aspartate(LOLA) versus Oral LOLA in patients with chronic liver disease(CLD) enduring overt Hepatic Encephalopathy(OHE). 40 CLD patients with OHE were randomly assigned IV or oral LOLA in a 1:1 ratio. Patients were graded for HE and monitored for serum ammonia levels from day 1 to day 5. The aim was to compare IV versus oral LOLA efficacy in HE grades improvement and its correlation with ammonia levels. The study was registered with clinical trials registry-India, CTRI/2020/12/029943. Baseline characteristics of patients in both groups were similar. The mean difference in ammonia levels from day 1 to day 5 was 55.4 ± 32.58 µmol/L in the IV LOLA group and 60.75 ± 13.82 µmol/L in the oral LOLA group (p = 0.511). Significant reductions in ammonia levels were observed from day 1 to day 5 within each group (p < 0.001). HE grade & ammonia correlated positively in both groups. LOLA, regardless of administration route, has demonstrated efficacy in OHE.

## Introduction

Cirrhosis, a prevailing disease of wide spectrum may result in central nervous system (CNS) alteration incorporating neurological or psychiatric realms. This detrimental continuum of clinic-pathological manifestations results from hyperammonemia, a congruous biochemical parameter observed in previously conducted studies^1^. Irrespective of inceptive occurrence either as minimal hepatic encephalopathy (MHE) or overt hepatic encephalopathy (OHE), disease invariably escalates to morbidity encompassing coma & stupor^2^.

Not only the symptoms, overt HE episodes & recurrences hamper the quality-of-life striking daily activities^3^. Among decompensated cirrhotic, incidence of HE reaches up to 50%^4^, symbolizing continual disease progression or deteriorating liver functions.

Aiming counteraction of this hyperammonemia, the linchpin of pharmaco-therapeutics relies either on one antibiotic, rifaximin^5^ or one non-absorbing disaccharide molecule, lactulose/lactitol^6^. These aids in minimizing intestinal ammonia production. Further advances in understanding of HE pathogenesis bring about l-ornithine-l-aspartate (LOLA) usage, capable of altering ammonia detoxification pathway^7^. Efficacy of LOLA in mitigating hyperammonemia & HE among cirrhotic has been proved invariably in multiple clinical studies conducted earlier.^8,9^

LOLA augments urea excretion by up-regulating intermediary pathways employing certain enzyme (carbamoyl phosphate & arginase) activities or substrate provisions. Overall, LOLA increases Krebs’ cycle turnover which further results in increasing plasma urea levels & simultaneously, plummeting serum ammonia^10^. Another intermediary metabolite, glutamine (Gln) plays important role in blood streaming ammonia, in its non-toxic form. LOLA indirectly expedites the glutamine synthesis & availability in blood via engaging glutamine synthase located in muscles & eventually, results in increased ammonia uptake^11^. After ingestion, LOLA disintegrates into constituent amino acids & then, actively absorbed in the small intestine apical layer.

Repeatedly proven in research, using LOLA in decompensated cirrhosis patients symptomatic with HE invariably exhibits beneficial effects & outcome irrespective of the route (enteral or parenteral) of administration^12^. However, these studies neither randomized efficiently nor shouldered arms properly as most of the observations used placebos. Another factor precipitating sparse availability of comparative data is the skewed focus of pharmacy driven evidences solely on manipulation of ammonia production.

Few studies advocated LOLA usage in HE management however, detailed analyses evaluating sequential ammonia levels & its correlative impingement on HE outcome still a matter of concern. Given this paucity, as a basis for planned research among cirrhosis suffering from HE, this study was conducted to ascertain the preferential route (Oral or IV) for LOLA effectiveness. Additional objective was to review the evidence base effect of LOLA in anti-HE modality stewardship.

## Methods

This is a single-centre randomized controlled study conducted in Sawai Man Singh Hospital, Jaipur, Rajasthan, India. The hypothesis(H0) considered for this study was significant difference in HE outcome & change in ammonia levels using LOLA as anti-HE modality either via Intravenous (IV) or Oral (PO) route, in a head-on comparison. The study was approved by the ethical committee of SMS Medical College, Jaipur & registered with Clinical Trials Registry-India, CTRI/2020/12/029943(21/12/2020). The study protocol was made in accordance with ethical guidelines of 1975 Helinski declaration.

All patients were enrolled after written and informed consent. All patients of cirrhosis from any cause, in the age group of 18 to 75 years with overt Hepatic encephalopathy (OHE) were included in the study. The exclusion criteria included terminally ill patients, those with advanced cardiac or pulmonary disease, chronic kidney failure (serum creatinine > 1.5 mg/dl), neurodegenerative disease (head injury, drug intoxication), major psychiatric illness, use of sedatives or antidepressants, pregnancy or breastfeeding, Hepatocellular carcinoma, acute on chronic liver failure, or any other identifiable cause for altered sensorium apart from hepatic encephalopathy.

**Figure 1:**
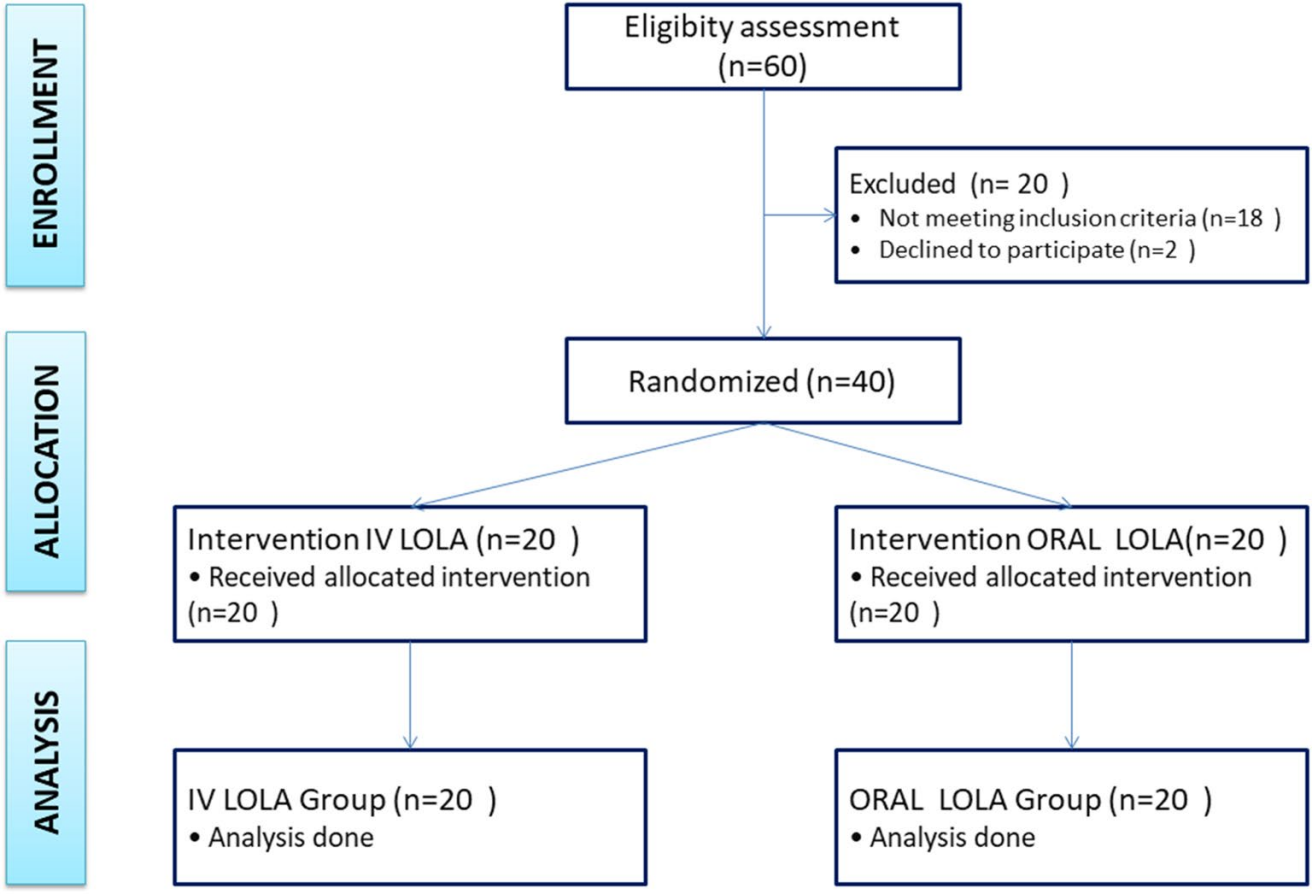
Flow chart of the study.

Sample size calculation was done based on the post-interventional alteration of ammonia levels in the study conducted by Keircheis et al.^13^ Assuming level of confidence at 95% with 90% power, targeted number of patients per group intended as > 14; group-wise sample size considered was 20 considering the fall-outs. The patients were randomly divided through a computer-generated random number into two groups; each group had 20 patients of OHE, as shown in Fig. 1. One group received oral LOLA in the form of 5 gm sachets every 4 hourly for 5 days. Those patients who were unable to swallow oral LOLA was administered the drug via nasogastric (Ryles’) tube. The other group of patients were given intravenous (IV) LOLA 30 gm over 24 h in 500 ml dextrose (D5%) infusion @20 ml/hr daily for 5 days. LOLA (IV or Oral) was given alongside standard anti-HE medications (lactulose & rifaximin) to both groups, along with other necessary treatments such as electrolyte correction, sepsis management with antibiotics, bleeding management, or other relevant interventions for comprehensive treatment. Computer generated randomization sequence was generated using on-site computer system in an open label fashion. Blinding was done using seemingly identical medications in both groups. Patients underwent routine investigations and daily venous blood serum ammonia evaluation. After recovery, patients underwent electroencephalogram prior to discharge.

### Statistical analysis

Categorical / Nominal variables were summarized as numbers and percentages and were analyzed using Chi square test. Continuous variables were summarized as mean and standard deviation and were analyzed using independent sample *t*-test for comparison between the two groups. Paired comparison within the same group at different time was done using paired t-test. Correlation between two continuous variables was assessed using Pearson correlation coefficient. A p-value ≤ 0.05 was taken as statistically significant. All statistical analysis was done using Epi info version 7.2.1.0 statistical software.

### Ethics declaration

The study was approved by the ethical committee of SMS medical college, Jaipur and registered with CTRI at No. CTRI/2020/12/029943 (21/12/2020). A written informed consent was obtained from the participants of the study. The study was performed in accordance with ethical guidelines of the 1975 Helinski. The participants were informed prior regarding right to refuse to participate in the study or to withdraw consent to participate at any time without reprisal. No animal was involved in this study.

## Results

Out of the total study population of 20 patients in each group, the majority were in age group of 40 to 55 years (70% versus 60%, oral versus IV group respectively). Overall, 65% patients were in this age bracket (40–55), as depicted in Table 1.

**Table 1:** Demographic parameters of study groups.

	LOLA (IV) (n = 20)	LOLA (ORAL) (n = 20)	p-value (95% C.I.)
Age (years), n (%)			
25–40	4 (20%)	5 (25%)	0.356
40–55	12 (60%)	14 (70%)	
55–70	4 (20%)	1 (5%)	
Male, n (%)	19 (95%)	20 (100%)	1.00
Female, n (%)	1 (5%)	0	
Alcohol consumers	15 (75%)	19 (95%)	0.182
Smoking	5 (25%)	4 (20%)	1.00
HBV	1 (5%)	2 (10%)	1.00
HCV	1 (5%)	0	1.00
Diabetes	4 (20%)	1 (5%)	0.342
HTN	4 (20%)	0	0.106
CTP class, n (%)			
A	0	0	0.715
B	4 (20%)	6 (30%)	
C	16 (80%)	14 (70%)	
MELD, n (%)			
30–39	1 (5%)	0	0.233
20–29	16 (80%)	13 (65%)	
10–19	3 (15%)	7 (35%)	
HE precipitants/possible causation, n (%)			
GI bleed	2	4	0.661
Diuretics	11	9	0.752
Sepsis	2	1	1.0
Paracentesis	6	8	0.741
Biochemical parameters			
Hb (g/dl)	9.2 ± 2.2	8.79 ± 2.82	0.611
WBC	8.23 ± 3.57	8.23 ± 4.24	1.000
INR	2.07 ± 0.75	1.79 ± 0.57	0.190
Urea (mg/dl)	41.65 ± 21.93	39.65 ± 19.18	0.761
Creatinine(mg/dl)	0.88 ± 0.33	0.93 ± 0.23	0.589
T. Bil (mg/dl)	4.79 ± 3.27	3.52 ± 2.53	0.182
SGOT (IU/L)	106.4 ± 133.25	81.45 ± 62.89	0.454
SGPT (IU/L)	55.2 ± 96.47	32.05 ± 15.59	0.296
ALP (IU/L)	84.8 ± 45.96	122.05 ± 79.83	0.078
Protein (mg/dl)	6.33 ± 1.04	6.2 ± 0.92	0.689
Albumin (mg/dl)	2.72 ± 0.35	2.68 ± 0.5	0.744
Sodium (meq/L)	132.15 ± 7.29	132.95 ± 4.64	0.681
Potassium (meq/L)	3.73 ± 0.69	4.12 ± 0.5	0.048

Among the oral group, all were males while in the IV group, 1 was female and 19 were males (100% versus 95%). Overall in this study, 97.5% patients were males.

A history of significant alcohol consumption was seen in 15(75%) patients in the IV LOLA group and in 19(95%) patients in the Oral LOLA group. Alcohol significance considered was equivalent to consuming 28–42 gm per day for a period of 10 years.

Seven patients in the IV group and five patients in the oral group had consumed alcohol within the six months prior to study enrollment. 25% patients in the IV LOLA group were chronic smokers while in the oral LOLA group 20% patients were chronic smokers. Etiological demographics suggested a prevailing share of ethanol as mere 5% in the IV LOLA group were HBV positive as compared to 10% in the Oral LOLA group. Similarly, only 5% in the IV LOLA group were HCV positive.

Co-morbidities among patients at baseline were collated & analyzed. 20% patients in the IV LOLA group were having diabetes (as per ADA guidelines)^14^ as compared to 5% in the oral LOLA group. 20% in the IV LOLA group were diagnosed having hypertension as compared to null prevalence among oral LOLA group.

Patients who were having underlying decompensated liver disease, further subjected to categorization according to the child-turcot-pugg (CTP) classification^15^. It was found that none of the patients in either group was of CTP-A class. 20% in the IV LOLA group belonged to CTP class B as compared to 30% in the oral LOLA group. 80% patients in the IV LOLA group belonged to CTP class C as compared to 70% in the oral LOLA group. Overall, in the study, 25% patients belonged to CTP-B class while 75% belonged to CTP-C.

Patients in the study were also classified according to MELD^16^ scores into three groups i.e., 10–19, 20–29 and 30–39. 15% in the IV LOLA group had MELD score of 10–19 as compared to 35% in the Oral LOLA group. 80% patients in the IV LOLA group had MELD score of 20–29 as compared to 65% in the Oral LOLA group. 5% in the IV LOLA group had MELD score of 30–39 versus none in oral LOLA group. Overall in the study, 25, 72.5 and 2.5% had MELD scores of 10–19, 20–29 and 30–39, respectively.

Comparison of biochemical parameters among study groups at baseline, revealed that the mean (± SD) hemoglobin values were 9.2 ± 2.2 gm% in the IV LOLA group as compared to 8.79 ± 2.82 gm% in the Oral LOLA group. Also, the mean total leukocyte count levels were 8.23 ± 3.57 × 10^3^/mm^3^ in the IV LOLA group as compared to 8.23 ± 4.24 × 10^3^/mm^3^ in the Oral LOLA group. The mean INR level was 2.07 ± 0.75 in the IV LOLA group as compared to 1.79 ± 0.57 in the Oral LOLA group.

Comparison of Renal function tests among study groups revealed that the mean blood urea values were 41.65 ± 21.93 mg/dl in the IV LOLA group and 39.65 ± 19.18 mg/dl in the Oral LOLA group. The mean serum creatinine levels were 0.88 ± 0.33 mg/dl in the IV LOLA group and 0.93 ± 0.23 mg/dl in the Oral LOLA group.

Analyzing liver function tests among study groups revealed that the mean serum bilirubin level was 4.79 ± 3.27 mg/dl in the IV LOLA group and 3.52 ± 2.53 mg/dl in the Oral LOLA group. The mean SGOT levels were 106.4 ± 133.25 U/L in the IV LOLA group and 81.45 ± 62.89 U/L in the Oral LOLA group while the mean SGPT levels were 55.2 ± 96.47 U/L in the IV LOLA group and 32.05 ± 15.59 U/L in the Oral LOLA group. The mean ALP level was 84.8 ± 45.96 U/L in the IV LOLA group and 122.05 ± 79.83 U/L in the Oral LOLA group. Though the mean serum total protein level was 6.33 ± 1.04 g/L in the IV LOLA group and 6.2 ± 0.92 g/L in the Oral LOLA group, the mean serum albumin level was 2.72 ± 0.35 g/L in the IV LOLA group and 2.68 ± 0.5 g/L in the Oral LOLA group.

Comparison of blood electrolytes among study groups revealed that the mean serum sodium was 132.15 ± 7.29 meq/L in the IV LOLA group and 132.95 ± 4.64 meq/L in the Oral LOLA group while the mean serum potassium was 3.73 ± 0.69 meq/L in the IV LOLA group and 4.12 ± 0.5 meq/L in the Oral LOLA group. Numerical values incorporating biochemistry aided in evaluation of precipitating or contributory factors for HE worsening, so that timely intervention can be done.

### Outcomes

**Figure 2:**
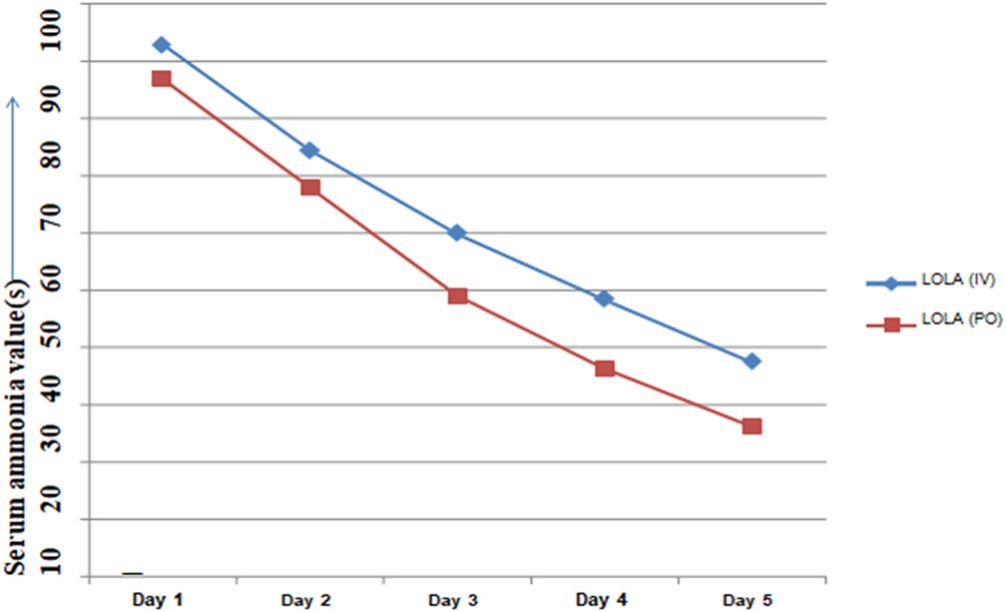
Line graph: day-wise linearity of serum ammonia level in both groups.

**Figure 3:**
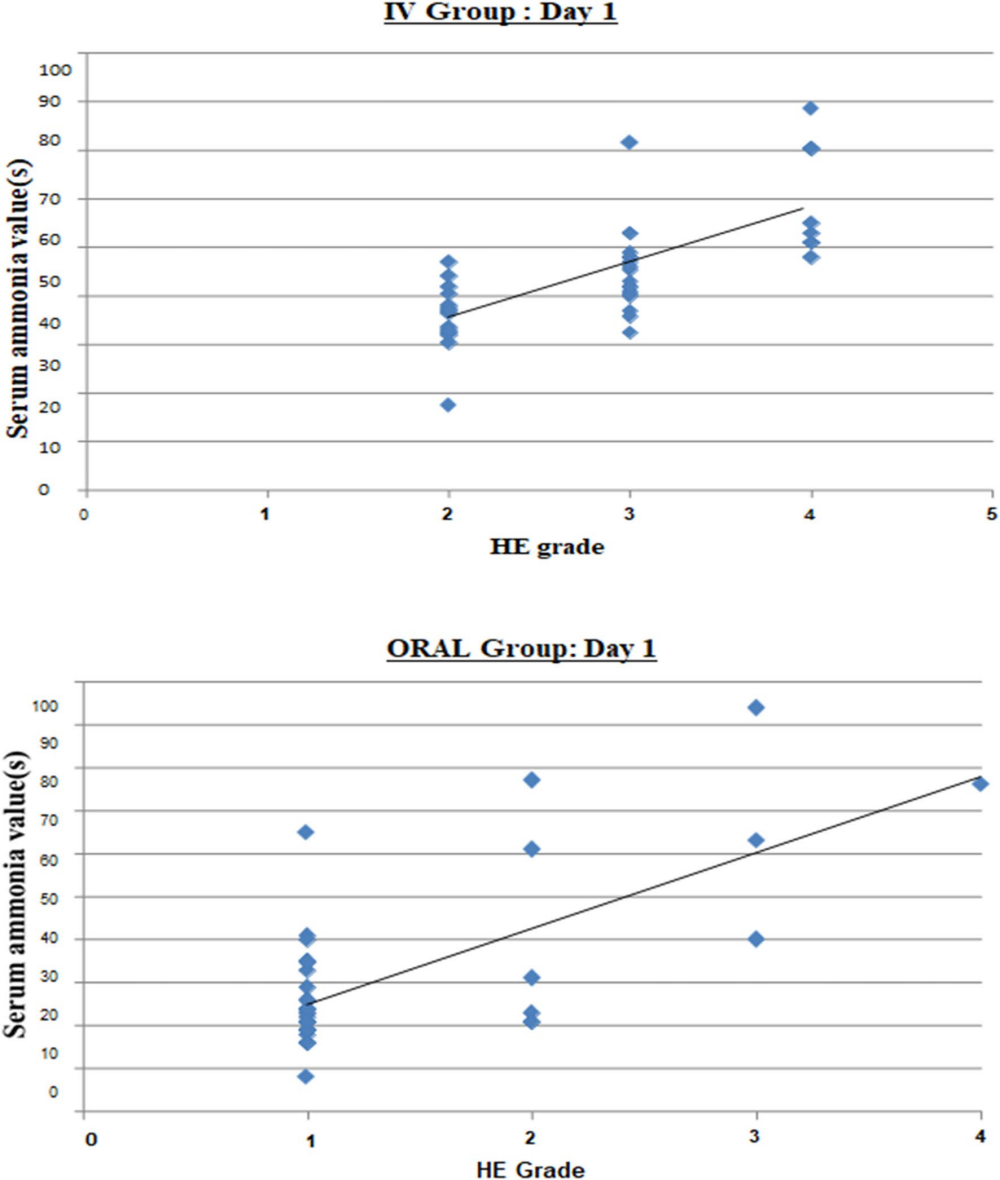
Scatter plot: HE grade & ammonia level correlation in both groups.

On comparing the baseline serum ammonia levels among study groups, it was found that the day-wise mean serum ammonia levels in individual groups decline continually in a linear pattern. This linear decline in serum ammonia levels in both groups has been shown with line graph, as in Fig. 2. Respective difference in mean ammonia values from day 1 to day 5 were 55.4 ± 32.58 umol/L and 60.75 ± 13.82 umol/L among IV & Oral LOLA group, a non-significant alteration when compared in-between groups (p = 0.511). However, the p-value showed significance (< 0.001) in comparative analysis of subsequent change in ammonia levels within same group i.e., from Day 1 to 5 as shown in Table 2. Scatter plots also displayed correlation between HE grade & ammonia levels in both groups, as in Fig. 3.

**Table 2:** Serum ammonia (NH_3_) levels among study groups.

	LOLA (IV)	LOLA (ORAL)	P-value (95% C.I.)
Day 1	92.65 ± 28.81	86.75 ± 19.11	0.450
Day 2	74.2 ± 29.89	67.8 ± 20.8	0.437
Day 3	59.6 ± 25.29	48.85 ± 19.93	0.144
Day 4	48.2 ± 26.03	36.1 ± 15.52	0.082
Day 5	37.25 ± 23.08	26 ± 13.12	0.066
∆NH_3_ (day 1-Day 5)	55.4 ± 32.58	60.75 ± 13.82	0.511
∆NH_3_ (within group)	< 0.001 (S)	< 0.001 (S)

Study subjects were additionally sorted into hepatic encephalopathy grades 1 to 4 (as per West Haven criteria)^17^ as shown in the Table 3. Correlative analysis of both groups to extrapolate linearity between ammonia levels & HE grade suggested Pearson’s coefficient value (r) as 0.702 (p < 0.001) and 0.682 (p < 0.001) on day 1 & 5, respectively (Table 4). Thus, vouched for significant positive correlation.

**Table 3:** Hepatic encephalopathy grading (day-wise) among study groups.

Day	HE grade	LOLA (IV)	LOLA (ORAL)	P-value (95% C.I.)
Day 1	II	6 (30%)	8 (40%)	0.675
	III	9 (45%)	9 (45%)	
	IV	5 (25%)	3 (15%)	
Day 2	I	2 (10%)	2 (10%)	1.000
	II	10 (50%)	11 (55%)	
	III	5 (25%)	6 (30%)	
	IV	3 (15%)	1 (5%)	
Day 3	I	7 (35%)	8 (40%)	0.837
	II	9 (45%)	7 (35%)	
	III	3 (15%)	5 (25%)	
	IV	1 (5%)	0	
Day 4	I	14 (70%)	12 (60%)	0.164
	II	3 (15%)	7 (35%)	
	III	3 (15%)	0	
	IV	0	1 (5%)	
Day 5	I	15 (75%)	15 (75%)	0.264
	II	2 (10%)	4 (20%)	
	III	3 (15%)	0	
	IV	0	1 (5%)

**Table 4:** Correlation between ammonia level and HE (IV versus oral group).

	r (correlation coefficient)	P value (95% C.I.)
Day 1	0.702	< 0.001 (S)
Day 5	0.682	< 0.001 (S)

## Discussion

This study on patients with cirrhosis analyzed our postulated hypothesis (H0) on the basis of results obtained regarding the efficacy of LOLA as indicated in improvement of mental state/HE and lowering of blood ammonia when administered, IV versus Oral route.

There exist quite a few studies evaluating the efficacy of LOLA in HE improvement & curtailing ammonia levels. Recently published, a study conducted by Sidhu et al. compared IV LOLA with placebo in assessing hyperammonemia reduction & mental state improvement^18^. Similarly, studies conducted by Abid et al., Schmid et al., Chen et al. & Kircheis et al. compared IV LOLA with placebo or standard anti-HE measures unanimously proved the efficacy & non-inferiority of LOLA in HE management^13,19,20^. Beside, some studies compared the usefulness of Oral LOLA routes versus placebo or Rifaximin as conducted by Alvares-da-Silva et al., Sharma et al., Mittal et al. & Stauch et al^21–24^. These publications also advocated the efficacy & superiority of LOLA as an anti-HE measure. Butterworth et al. conducted a systematic review in 2018 to independently assess the efficacy of LOLA either in IV or Oral form & further validated its ammonia lowering & mental status improving effect in all HE as well OHE groups^2^. Nonetheless, current AASLD-EASL guidelines^21^ on HE management denied affirmative nod regarding LOLA usage in Oral form ascribing the results of RCT conducted by Kircheis et al. in which IV usage proved efficacious with gastrointestinal adversities in some patients^13^. This underscores the need for reviewing & analyzing LOLA efficacy in Oral versus IV routes in one-to-one comparative format, as done in this RCT.

Head-to-head trials comparing the efficacy of LOLA with other ammonia-lowering agents (lactulose, rifaximin or probiotics) have consistently shown that LOLA is equivalent and, in some cases, superior to alternative agents although, majority of these studies included MHE patients considering ethical perspective^22,25^. Studies are still inadequate recruiting OHE patients having severe grades (West Haven) to buttress conclusive evidence. Prevention therapeutics in MHE is an altogether different aspect which also needs mention.

Not only in mitigating ammonia, cerebral edema & HE manifestations, LOLA also aids in normalization of transaminases, bilirubin levels & coagulopathy parameters in cirrhosis^26^. Proposed physiologies involve augmented nitric oxide (NO) and glutathione (GSH) production discretely exerting micro-vascular dilatation & anti-oxidant effects. This indirectly results in improved hepatic functioning & hence, ammonia clearance. LOLA, when added to other anti-HE treatments, has been proven effective and should be used in therapy without hesitation. Moreover, beyond HE treatment, Horvath et al. recently observed LOLA’s positive impact on enhancing the microbiome and beneficial metabolomic changes, such as IGF-1 levels, in liver disease patients^27^.

Besides having supremacy in various individual trials, some systematic reviews & meta-analyses also advocated LOLA role in anti-HE armamentarium. 246 patients recruited from 5 RCTs suggested 3.22 folds higher recovery from HE after 7 days of therapy as compared to placebo^28^. Another high-quality (Jaded) meta-analysis involving 212 patients endorsed LOLA usage in OHE grade 1or 2, however no significant boost seen in MHE^29^. As compared to placebo, lactulose or probiotics aiming to improvise metal state, LOLA asserted higher & equal efficacy respectively; in an updated meta-analysis of 646 patients^30^. A recently published review in 2019 included 919 patients from 10 RCTs also favored LOLA for its effect on hyperammonemia & altered mental state^31^.

Database search targeting effectual route of LOLA administration for maximal outcome in HE happened to be a futile exercise attributing to the paucity of head-to-head comparative studies. An abstract presented in EASL meet 2018 advocated the superiority of Oral route over IV, although recruited MHE patients only^32^. This again brings out the obligation of conducting a study to evaluate the advantageous route for LOLA, be it enteral (PO) or parenteral (IV).

In this study, we compared therapeutic efficacy of Oral LOLA against IV LOLA as an adjunct to the conventional anti-HE drugs in cirrhosis decompensated with HE. Grades of HE (West Haven) showed a linear correlation with serum ammonia levels at all analytical time-points during this study, similar to available scientific data. Serum ammonia levels showed a steady falling curve over the period of 5 days during which LOLA was given with a significant change in absolute values i.e., day 1 to day 5, in each group (p < 0.001). However, comparing the mean change in ammonia levels in both groups resulted in p-value of 0.511. A similar trend of sequential improvement was seen in HE grade categorization as well, among the groups. The results point to formulate an alternate hypothesis (HA) as both formulation (IV & Oral) proved efficacious in achieving primary outcome but intergroup variations were not significant. Furthermore, since the efficacy demonstrated is comparable in reducing ammonia levels and improving HE grades, across groups, the author suggests the oral route as the preferred option due to its ease of administration and cost-effectiveness compared to the intravenous route, unless the latter is not feasible due to other clinical circumstances.

LOLA when used as a supplemental measure to other drugs (lactulose & rifaximin) infallibly, results in lesser recovery time & shorter hospital stay attenuating overall morbidity & mortality. Whether LOLA can be used as a stand-alone therapy or as a superior complementarity; still a matter of debate & further evaluation. Role of LOLA in prophylaxis & MHE has been proved in some studies however, not a targeted outcome in this study.

Major limitation of this study was small sample size & single-centric format. Treatment given was in adjunct to conventional therapeutics, hampering the assessment of efficacy as a sole agent. Duration of the treatment considered was of 5 days, abstracted from multiple previous studies with a variable range from 3 to 7 days. Though few studies advocate arterial blood samples^33^ as a better corroborative marker for hyperammonemia, venous blood sampling was used in this study accrediting technicalities. Even so, no major or life threatening adversities observed in the study, follow up data was not collated to ascertain long term outcomes.

## Conclusion

LOLA, a synthetic analogue containing two endogenous amino acids, has been proven time invariably concerning its benefaction in ameliorating hepatic encephalopathy. Results of this RCT comparing Oral with Intravenous (IV) LOLA in patients of chronic liver disease with overt hepatic encephalopathy showed a significant decrease in serum ammonia levels along with concomitant improvement in hepatic encephalopathy grades in both the groups when analyzed independently but the intergroup difference did not exhibit statistical significance. Future RCTs recruiting more number of patients are required to establish the difference correctly. Till then, based on the observations of this comparative study, LOLA whether Oral or IV can be used with similar efficacy to decrease serum ammonia levels and improves hepatic encephalopathy in select cases of chronic liver disease. However, oral route may be preferrable due to ease of administration & feasibility. LOLA should always be used in addition to other anti-HE proven efficacies as clear demarcating stewardships for its sole usage in OHE are still in developmental stages.

### Supplementary Information

Supplementary Information 1.

Supplementary Information 2.

Supplementary Information 3.

## Data Availability

The data that support the findings of this study are available from the corresponding author upon reasonable request.
