# Supplementary material for: Intravenous versus oral ‘l-ornithine-l-aspartate’ in overt hepatic encephalopathy: a randomized comparative study
Source: Sci Rep. 2024 May 24;14:11862. doi: 10.1038/s41598-024-62293-8 (PMC11126676; doi:10.1038/s41598-024-62293-8)
Supplement: Supplementary file 1 — Supplementary Information 1. [file 41598_2024_62293_MOESM1_ESM.docx]

**HIGHLIGHTS**

- HE episodes have a prognostic value in decompensated chronic liver disease.
- Lactulose & Rifaximin are conventional anti-HE measures.
- LOLA usage supplements the effectiveness of HE treatment employing its diverse metabolic pathways of ammonia detoxification & hepato-protective action.
- LOLA, irrespective of mode of administration has significant efficacy as an anti-HE drug however, comparative intergroup study for preferable route generate equivocal results.
