# Supplementary material for: Intravenous versus oral ‘l-ornithine-l-aspartate’ in overt hepatic encephalopathy: a randomized comparative study
Source: Sci Rep. 2024 May 24;14:11862. doi: 10.1038/s41598-024-62293-8 (PMC11126676; doi:10.1038/s41598-024-62293-8)
Supplement: Supplementary file 2 — Supplementary Information 2. [file 41598_2024_62293_MOESM2_ESM.docx]

**Intravenous versus Oral ‘L-Ornithine-L-Aspartate’ in Hepatic Encephalopathy: A randomized comparative study for superior route**

**AUTHORS**

***Ashok Jhajharia^1^, Shashank Singh, Sangeeta Jana, Prachis Ashdhir, Sandeep Nijhawan***

*Department of Gastroenterology, SMS Medical College & Hospital, Jaipur, Rajasthan, India*

**CORRESPONDENCE**

*drashokjhajharia@gmail.com*

**^1^Dr. Ashok Jhajharia,** Associate Professor, Department of Gastroenterology, SMS Medical College & Hospital, Jaipur-302004, India

**LAY SUMMARY**

Hepatic encephalopathy management should consist of using all available & efficacious therapeutic measures for timely and safe outcomes. Beside using time-proven conventional drugs like lactulose & rifaximin, some newer drugs also shown benefits. We, in this study have focused on L-ornithine-L-aspartate (LOLA), a drug that adjuncts anti-HE treatment. We ascertained its efficacy as an anti-HE measure & also tried to evaluate the preferred mode of administration for a superior result.
