# Supplementary material for: Intravenous versus oral ‘l-ornithine-l-aspartate’ in overt hepatic encephalopathy: a randomized comparative study
Source: Sci Rep. 2024 May 24;14:11862. doi: 10.1038/s41598-024-62293-8 (PMC11126676; doi:10.1038/s41598-024-62293-8)
Supplement: Supplementary file 3 — Supplementary Information 3. [file 41598_2024_62293_MOESM3_ESM.pdf]

Clinical Trial Details (PDF Generation Date :- Mon, 08 Jan 2024 00:52:41 GMT)

**CTRI Number**	CTRI/2020/12/029943 [Registered on: 21/12/2020] - **Trial Registered Prospectively**
**Last Modified On**	03/11/2022
**Post Graduate Thesis**	Yes
**Type of Trial**	Interventional
**Type of Study**	Drug
**Study Design**	Randomized, Parallel Group, Active Controlled Trial
**Public Title of Study**	Comparison of injectable L-Ornithine L -Aspartate versus oral L-Ornithine L -Aspartate for treatment of Cirrhosis patients with altered behavior.
**Scientific Title of Study**	Randomized comparative study to assess the efficacy of injectable L-Ornithine L -Aspartate versus oral L-Ornithine L -Aspartate for the treatment of overt hepatic encephalopathy in Cirrhosis

Secondary IDs if Any

Secondary ID	Identifier
NIL	NIL

Details of Principal Investigator or overall Trial Coordinator (multi-center study)

Details of Principal Investigator	
**Name**	Dr Ashok Jhajharia
**Designation**	Assistant Professor
**Affiliation**	SMS Medical College Jaipur
**Address**	Department of Gastroenterology Room No. 303, IIIrd Floor SMS Hospital, Jaipur Department of Gastroenterology Room No. 303, IIIrd Floor SMS Hospital, Jaipur<br>Jaipur<br>RAJASTHAN<br>302004<br>India
**Phone**	9414236572
**Fax**	01412518480
**Email**	drashokjhajharia@gmail.com

Details Contact Person (Scientific Query)

Details Contact Person (Scientific Query)	
**Name**	Dr Ashok Jhajharia
**Designation**	Assistant Professor
**Affiliation**	SMS Medical College Jaipur
**Address**	Department of Gastroenterology Room No. 303, IIIrd Floor SMS Hospital, Jaipur Department of Gastroenterology Room No. 303, IIIrd Floor SMS Hospital, Jaipur<br>Jaipur<br>RAJASTHAN<br>302004<br>India
**Phone**	9414236572
**Fax**	01412518480
**Email**	drashokjhajharia@gmail.com

Details Contact Person (Public Query)

Details Contact Person (Public Query)	
**Name**	Rishabh Gupta
**Designation**	Senior Resident
**Affiliation**	SMS Medical College Jaipur
**Address**	Department of Gastroenterology Room No. 303, IIIrd Floor SMS Hospital, Jaipur Department of Gastroenterology Room No. 303, IIIrd Floor SMS Hospital, Jaipur<br>Jaipur<br>RAJASTHAN

	302004<br>India
**Phone**	9588248715
**Fax**	01412518480
**Email**	rishabh1313@gmail.com

Source of Monetary or Material Support

Source of Monetary or Material Support
> SMS Medical College and Attached Group of Hospital, Jaipur

Primary Sponsor

Primary Sponsor Details	
**Name**	BSBY Scheme Govt of Rajasthan
**Address**	Department of Gastroenterology Room No. 303, IIIrd Floor SMS Hospital, Jaipur
**Type of Sponsor**	Government medical college

Details of Secondary Sponsor

Name	Address
Rajasthan Medicare Relief Society	SMS Hospital Jaipur

Countries of Recruitment

List of Countries
India

Sites of Study

Name of Principal Investigator	Name of Site	Site Address	Phone/Fax/Email
Dr Ashok Jhajharia	SMS Medical College Jaipur	Department of Gastroenterology Room No. 303, IIIrd Floor SMS Hospital, Jaipur<br>Jaipur<br>RAJASTHAN	9414236572<br>01412518480<br>drashokjhajharia@gmail.com

Details of Ethics Committee

Name of Committee	Approval Status	Date of Approval	Is Independent Ethics Committee?
office of ethics committee sms medical college jaipur	Approved	29/10/2020	No

Regulatory Clearance Status from DCGI

Status	Date
Not Applicable	No Date Specified

Health Condition / Problems Studied

Health Type	Condition
Patients	Hepatic fibrosis with hepatic sclerosis

Intervention / Comparator Agent

Type	Name	Details
Comparator Agent	Injectable Lola Versus Oral Lola	Injectable l-ornithine aspartate Dose 30 Gm Intravenous dissolved in 5% Dextrose fluid given in one arm compared to oral l-ornithine aspartate Dose of 5Gm 4 hourly with total Dose of 30 Gm in another arm in patients of overt Hepatic Encephalopathy
Intervention	drug by oral or intravenous route	in one group LOLA is given oral and in other group it is administered intravenously

Inclusion Criteria

Inclusion Criteria	
**Age From**	18.00 Year(s)
**Age To**	75.00 Year(s)
**Gender**	Both

**Details**	1. Cirrhosis<br>2. Age 18-75 years<br>3. Overt HE (Grade 2-4)

Exclusion Criteria

Exclusion Criteria	
**Details**	1. Terminally ill patients<br>2. Advanced cardiac or pulmonary disease<br>3. Underlying chronic renal failure or acute kidney injury with serum creatinine > 1.5 mg/dl<br>4. Neurodegenerative disease or major psychiatric illness<br>5.Use of sedatives or antidepressants<br>6. Pregnancy or breast-feeding<br>7.HCC<br>8. ACLF

**Method of Generating Random Sequence**	Computer generated randomization
**Method of Concealment**	On-site computer system
**Blinding/Masking**	Open Label

Primary Outcome

Outcome	Timepoints
Mental State Grade	5 Days of Treatment

Secondary Outcome

Outcome	Timepoints
1.Change in blood ammonia levels<br>2. Rate of recovery from HE<br>3. Length of hospital stay.	1.Change in blood ammonia levels at day five compared to day one.<br>2. Rate of recovery from HE<br>3. Length of hospital stay.

**Target Sample Size**	**Total Sample Size=**40<br>**Sample Size from India=**40<br>**Final Enrollment numbers achieved (Total)=**Applicable only for Completed/Terminated trials<br>**Final Enrollment numbers achieved (India)=**Applicable only for Completed/Terminated trials
**Phase of Trial**	N/A
**Date of First Enrollment (India)**	25/12/2020
**Date of First Enrollment (Global)**	No Date Specified
**Estimated Duration of Trial**	**Years=**0<br>**Months=**6<br>**Days=**0
**Recruitment Status of Trial (Global)**	Not Applicable
**Recruitment Status of Trial (India)**	Closed to Recruitment of Participants
**Publication Details**	Nil
**Brief Summary**	It will be a prospective, randomized controlled comparative study to evaluate the efficacy of injectable lola (l - ornithine l - aspartate) versus oral lola for the treatment of overt hepatic encephalopathy in cirrhosis. All the patients fulfilling the inclusion criteria will be randomized into two groups of 25 each and will be given the above drugs for 5 days. Standard care treatment will be followed, primary and secondary outcomes will be noted.<br><br>Till now there has been not trial comparing the efficacy of oral lola versus injectable lola for overt hepatic encephalopathy in patients of cirrhosis.
